# Risk of cervical pre-cancer and cancer in women with multiple sclerosis exposed to high efficacy disease modifying therapies

**DOI:** 10.3389/fneur.2023.1119660

**Published:** 2023-02-10

**Authors:** Francesca Bridge, Julia M. L. Brotherton, Yi Foong, Helmut Butzkueven, Vilija G. Jokubaitis, Anneke Van der Walt

**Affiliations:** ^1^Department of Neuroscience, Central Clinical School, Monash University, Melbourne, VIC, Australia; ^2^Department of Neurology, Alfred Health, Melbourne, VIC, Australia; ^3^Australian Centre for the Prevention of Cervical Cancer (Formerly Victorian Cytology Service), Carlton South, VIC, Australia; ^4^Centre for Epidemiology and Biostatistics, Melbourne School of Population and Global Health, University of Melbourne, Melbourne, VIC, Australia; ^5^Department of Neurosciences, Eastern Health, Melbourne, VIC, Australia

**Keywords:** cervical cancer, disease modifying therapy (DMT), autoimmune disease (AID), multiple sclerosis (MS), human papilloma virus (HPV)

## Abstract

There is a growing need to better understand the risk of malignancy in the multiple sclerosis (MS) population, particularly given the relatively recent and widespread introduction of immunomodulating disease modifying therapies (DMTs). Multiple sclerosis disproportionately affects women, and the risk of gynecological malignancies, specifically cervical pre-cancer and cancer, are of particular concern. The causal relationship between persistent human papillomavirus (HPV) infection and cervical cancer has been definitively established. To date, there is limited data on the effect of MS DMTs on the risk of persistent HPV infection and subsequent progression to cervical pre-cancer and cancer. This review evaluates the risk of cervical pre-cancer and cancer in women with MS, including the risk conferred by DMTs. We examine additional factors, specific to the MS population, that alter the risk of developing cervical cancer including participation in HPV vaccination and cervical screening programs.

## 1. Introduction

There is growing need to better understand the risk of cancer in the multiple sclerosis (MS) population, particularly with the recent and widespread introduction of highly effective immunomodulatory or immunosuppressive disease modifying therapies (DMTs) (1, 2). MS is a chronic inflammatory autoimmune disease of the central nervous system that is three times more prevalent in women and usually diagnosed between the ages of 20–40 years (3). Treatment with DMTs is usually commenced at diagnosis and continued life-long, leading to significant long-term exposure (4).

A recent scoping review highlighted gynecological cancer risk, including cervical cancer, as an important knowledge gap in the MS literature (1). The risk of cervical cancer may be altered in women with MS (wwMS). It remains unclear whether this is the result of the autoimmune condition or secondary to the DMTs used in the treatment of MS. Additionally, wwMS may be underrepresented in primary and secondary prevention programs (5–9), further contributing to the risk of cancer in this vulnerable patient population.

**Figure 1:**
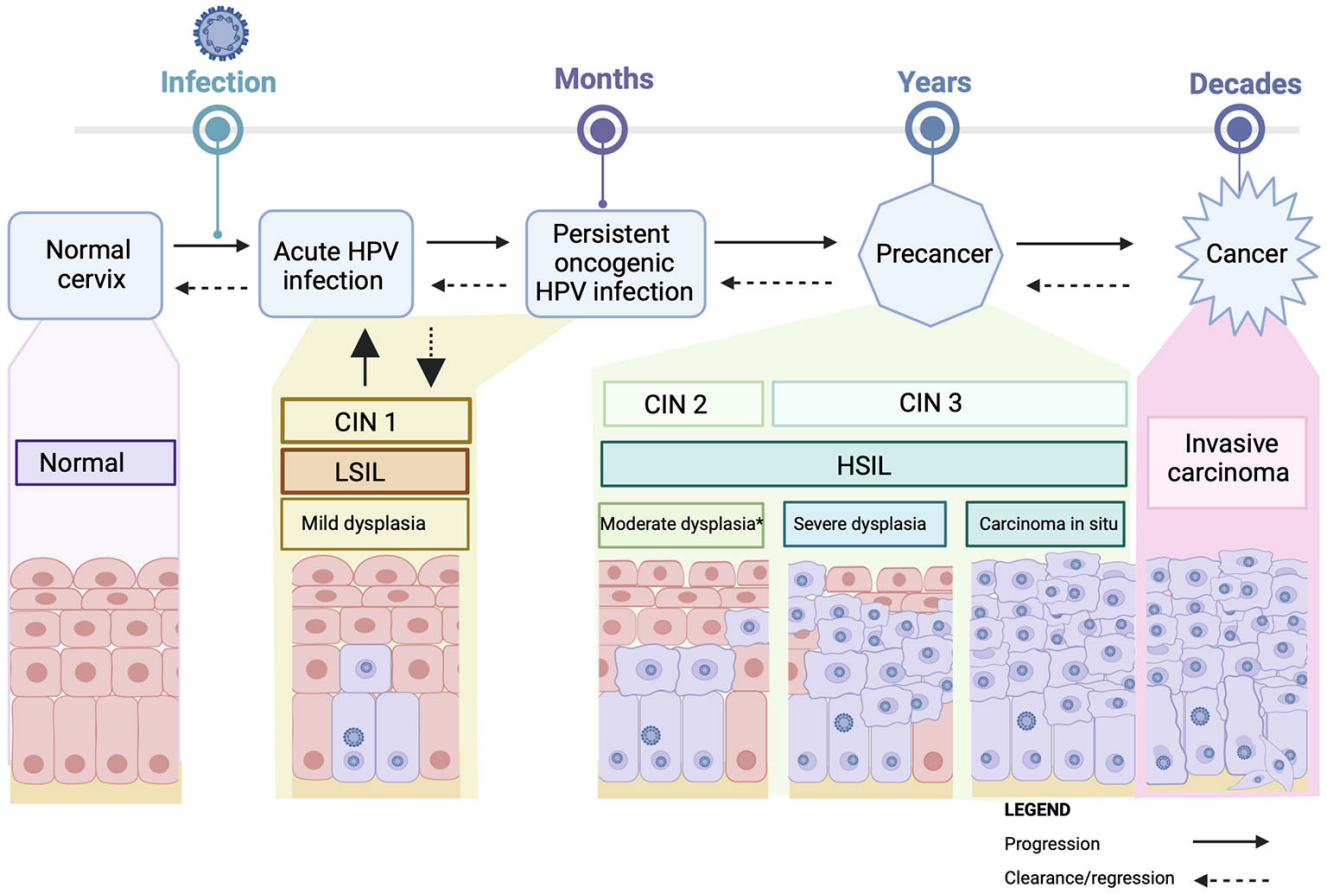
Cervical carcinogenesis. Acute Infection with HPV may cause mild cervical abnormalities [CIN I (mild dysplasia/LSIL)], which usually clear spontaneously. Persistent infection with oncogenic HPV can result in cervical pre-cancerous lesions [CIN 2 (moderate dysplasia), CIN3 (severe dysplasia or carcinoma *in situ*/HSIL)] which can progress over time to invasive cancer. Progression is not inevitable, with regression possible at any stage. ^*^sometimes acute infections/infections with lower risk HPV types can produce an appearance that is identified as moderate disease (CIN2). CIN2 is a heterogeneous entity likely comprising “severe CIN1 cases” and “mild CIN2” cases rather than a single disease state with a uniform prognosis. HPV, human papillomavirus; CIN, cervical intraepithelial neoplasia; LSIL, low-grade squamous intraepithelial lesions; HSIL, high-grade squamous intraepithelial lesions. Created with BioRender.com.

Almost all cervical cancer is due to an underlying persistent infection with oncogenic types of human papillomavirus (HPV) (10, 11), a very common double stranded DNA virus, that is transmitted *via* sexual contact (12–14). HPV is usually cleared by the immune system without symptoms within 1–2 years. Persistent infection with one of the 13 oncogenic HPV types (including the most oncogenic types, HPV 16 and HPV 18, which are associated with 70% of cervical cancers globally) increases the risk of developing cervical pre-cancerous abnormalities. Cervical pre-cancerous abnormalities are classified as cervical intraepithelial neoplasia (CIN) grade 2 or 3 (CIN3 is synonymous with carcinoma in situ), or adenocarcinoma in situ (AIS). These abnormalities also referred to as High-grade squamous intraepithelial lesions (HSIL). Pre-cancerous lesions are at risk of progression to cancer of the cervix (see Figure 1) (12, 13). Co-factors for the development of cervical cancer in the presence of persistent oncogenic HPV include smoking, high parity, oral contraceptive use, and immunocompromise. An intact immune system is necessary for adequate clearance of HPV. HPV clearance is impaired in patients who are severely immunocompromised, including patients with human immunodeficiency virus (HIV) or acquired immunodeficiency syndrome (AIDS), solid-organ transplant recipients and some autoimmune conditions (4, 15–17). The immunocompromised population are at risk of infection with multiple HPV types along with a greater diversity of HPV types (18–20).

Here we review the literature relating to the risk of cervical cancer in people with a cervix, hereby referred to as women with MS. We consider the role that immunotherapies play in altering this risk. Furthermore, we explore other factors, specific to the MS population, that may impede access to HPV vaccination and cervical screening programs and further increase patients' risk of developing cervical cancer.

## 2. Methodology

### 2.1. Literature search

We searched the PubMed database for peer-reviewed articles published in English from 1990 through October 2022 on multiple sclerosis and cervical cancer risk, using the following search strategy:

((((((“multiple sclerosis”[MeSH Terms] OR (“multiple”[All Fields] AND “sclerosis”[All Fields]) OR “multiple sclerosis”[All Fields]) AND (“uterine cervical neoplasms”[MeSH Terms] OR (“uterine”[All Fields] AND “cervical”[All Fields] AND “neoplasms”[All Fields]) OR “uterine cervical neoplasms”[All Fields] OR (“cervical”[All Fields] AND “cancer”[All Fields]) OR “cervical cancer”[All Fields])) AND (fft[Filter])) OR (((“neoplasms”[MeSH Major Topic] OR “uterine cervical neoplasms”[MeSH Major Topic]) AND “multiple sclerosis”[MeSH Major Topic]) AND ((fft[Filter]) OR ((viral)) AND (cancer)) AND (multiple sclerosis)) AND (1990:2022[pdat]))))

In addition, we hand-searched reference lists from the articles identified by the search and key review articles. We also reviewed the pivotal pharmaceutical trials for each DMT.

### 2.2. Selection criteria

We reviewed titles, abstracts to identify studies examining the risk of cervical abnormalities in wwMS, including the risk conferred by DMTs.

## 3. Risk of cervical cancer in wwMS

There is limited literature on the risk of cervical abnormalities and cancer in wwMS (see Table 1). The reported incidence of cervical cancer in wwMS ranges from 0.1–1.1 per 1,000 person years (21, 23, 24, 27, 28, 36). The heterogeneity in incidence estimates is likely due to the differences in the way the MS population was identified, study sample sizes, methods for reporting cancers, observation periods, and variation in DMT use across these studies (36, 37).

**Table 1:** Summary of studies that evaluate cervical cancer risk in patients with multiple sclerosis.

**Authors**	**Type of study**	**Country**	**MS sample size**	**Index period**	**Data source for MS patients**	**Results risk of cervical cancer**		**Interpretation**
						***N*** **(%)**	**SIR/HR/AHR**	
**Studies which found decreased risk cervical abnormalities**								
Moller et al. (21)	Cohort study	Denmark	5,359 MS patients 3,165 women	1977–1987	Registry data linkage: Danish hospital discharge register, Danish cancer register	Cervical cancer MS 5	RR 0.9, *p* ≤ 0.05	Risk of cervical cancer reduced in wwMS
Moisset et al. (22)	Case-control study	France	1,107 MS patients 1,568 controls	2014–2015	“MS patients' network in Auvergne” association members	Gynecological cancers MS 13 (1.17%) Controls 28 (2.93%)	NR	Risk of cervical cancer not increased in wwMS
**Studies which found cervical abnormality risk same as general population**								
Nielsen et al. (23)	Cohort study	Denmark	11,817 MS patients 7,188 women	1968–1997	Registry data linkage (Danish MS register, Danish cancer register)	Cervical cancer 40	SIR 1.11 (0.81–1.51)	No difference in cervical cancer incidence in wwMS
Bahmanyar et al. (24)	Cohort study	Sweden	20,276 MS (13,218 wwMS) 203,951 non-MS controls (132,638 women)	1958–2005	Data linkage registry data: Swedish MS register and national inpatient register), patients with MS matched to age, sex, area controls	Cervical cancer 63	HR 0.83 (95% CI 0.64-1.07)	No difference in cervical cancer risk in wwMS compared with controls
Fois et al. (25)	Observational study	United Kingdom	4,250 MS patients 2,812 women	1963–1999	Oxford record linkage study (ORLS)	Cervical cancer 6	Adjusted rate ratio 1.3 (95% CI 0.5–2.8, *p* = 0.75)	No increased risk of cervical cancer in wwMS
Lebrun et al. (26)	Descriptive study	France	20,993 MS patients 15,220 women	1995–2009	Registry data: European database for MS, French national cancer registry	Gynecological (ovarian, cervix, uterine) cancer 28	SIR 1.2 (0.8–1.9)	No difference in cervical cancer incidence in wwMS
Kingwell et al. (27)	Retrospective cohort study	Canada	6,820 MS patients 4,998 women	1980–2004	Registry data linkage: British Columbia MS database, British Columbia cancer registry, British Columbia ministry of health's registration and premium billing files, British Columbia vital statistics death database	Cervical cancer 8	SIR 0.84 (0.36–1.65)	No difference in cervical cancer incidence in wwMS compared with controls
Hemminki et al. (28)	Observational study	Sweden	185,014 wwMS	1964 (some regions) 1986–2008	Linkage of national datasets: Swedish hospital discharge register, Swedish cancer registry	Cervical cancer 18 Cervical cancer deaths 8	Cervical cancer SIR 0.92 (95%CI 0.54–1.45) Cervical cancer deaths HR 1.81 (95% CI 0.91–3.62)	Incidence of cervical cancer and cervical cancer deaths in wwMS the same as the general population
Dugué et al. (29)	Cohort study	Denmark	14,403 wwMS	1977–2010	Registry data linkage: Danish national patient register, Danish national prescription registry, Danish cancer register	Cervical cancer 1977–2010: 46 1995–2010: 28	1977–2010: SIR 1.2 (0.9–1.6) 1995–2010: SIR 1.2 (0.8-1.8)	No difference in cervical cancer incidence in wwMS
Ajdacic-Gross et al. (30)	Case-control study	Switzerland	5,489 MS patients number of women NR	1969–2007	Data linkage from medical records and hospital coding	Cervical cancer 20	SMR 1.11 (chi2 lCI-uCI 1.03–1.46, *p* > 0.1)	Cervical cancer mortality not increased in the MS population
Hongell et al. (31)	Case-control study	Finland	1,974 MS patients 10,740 controls	2004–2012	Hospital administrative data	Cervical cancer MS 1 (0.1%) Controls 5 (0.0%)	OR 2.0 (95% CI 0.2–16.4, *p* = 0.519)	No increased risk of cervical cancer in MS population
Grytten et al. (32)	Prospective cohort study	Norway	6,883 MS patients (4597 wwMS) 27,919 population controls (25,265 women)	1952–2016	Registry data linkage: Norwegian MS registry, cancer registry of Norway	Female genital organ cancer MS 94 (12.1%) Controls 459 (11.4%)	HR 1.18 (0.94–1.47)	No increased risk of female genital organ cancer in wwMS compared to controls
Foster et al. (4)	Retrospective cohort study	Australia	1,426 wwMS 985,383 non-MS controls	2000–2013	Data linkage: Victorian emergency minimum dataset, victorian cervical cytology register	High grade histology 40 High grade cytology 77 Low grade cytology 251	High-grade histological abnormalities: 3.07 vs. 3.76 per 1,000 person-years, AHR = 0.78, *p* = 0.124 High-grade cytological abnormalities (5.98) vs. 6.26 per 1,000 person-years, AHR = 0.98, *p* = 0.836 Low-grade histological abnormalities: 20.45 vs. 19.99 per 1,000 person-years, AHR = 1.10, *p* = 0.139	No difference in risk of cervical abnormalities (high or low-grade) in wwMS compared with controls
Johnson et al. (33)	Observational study	North America	7,277 MS patients 7,277 controls	1997-NR	Health record database used to identify cases of MS and age, race, and gender matched controls	Anal/vaginal/cervical cancer MS 11 (0.2%) Controls 13 (0.2%)	NR	No difference in cervical cancer incidence in wwMS
Grytten et al. (34)	Cohort study	Norway	6,949 MS patients (4,638 wwMS) 37,922 controls (2,513 women)	1953–2017	Registry data linkage	Female genital organ cancer 1953–1995: 6 1996–2017: 68	Female genital organ cancer: 1953–1995: IRR 0.78 (95% CI 0.48–1.27) 1996–2017: IRR 1.40 (95% CI 1.09–1.80, *p* < 0.05)	1953–1995 no difference in cervical cancer frequency in MS patients 1996–2017 increased frequency of female genital organ cancer in wwMS
Marrie et al. (35)	Retrospective matched cohort study	Canada	53,983 MS cases 269,915 controls	1998–2017	Population-based administrative databases: manitoba population research data repository, institute for clinical evaluative sciences (ICES)	NR	Cervical cancer crude IRR 1998–2007 0.85 (95% CI 0.5–1.45) 2008–2017 0.85 (95% CI 0.55–1.31) Age-standardized IRR 1998–2007 0.92 (0.52–1.63) 2008–2017 0.84 (0.53–1.33)	Cervical cancer incidence not increased in the MS population
**Studies which found increased risk cervical abnormalities**								
Grytten et al. (34)	Cohort study	Norway	6,949 MS patients (4,638 wwMS) 37,922 controls (2,513 women)	1953–2017	Registry data linkage: Norwegian MS registry, cancer registry of Norway	Female genital organ cancer 953–1995: 6, 1996–2017: 68	Female genital organ cancer: 1953–1995: IRR 0.78 (95% CI 0.48–1.27) 1996–2017: IRR 1.40 (95% CI 1.09–1.80, *p* < 0.05)	1953–1995 no difference in cervical cancer frequency in MS patients 1996–2017 Increased frequency of female genital organ cancer in MS patients

AHR, adjusted hazard ratio; CI, confidence interval; DMT, disease modifying therapy; HR, hazard ratio; IRR, incidence rate ratio; MS, multiple sclerosis; NR, not reported; OR, odds ratio; RR, relapse rate; SIR, standardized incidence ratio; SMR, standardized mortality ratio; wwMS, women with MS.

Two retrospective observational studies, one Finnish and the other Australian, evaluated the risk of cervical abnormalities in wwMS (4, 31). Women with MS were found not to be at increased risk of cervical cancer or high-grade histological and cytological abnormalities, respectively. However, it is important to consider important limitations of both studies which could impact their generalizability. Firstly, the cohorts were identified through hospital administration data (4, 31). Hospitalization is atypical for the MS population, and could represent patients with more severe disease, or significant medical comorbidities. Secondly, neither study examined the impact of DMT use, either due to lack of information, or insufficient power (4, 31).

The finding that cervical cancer risk in the MS population is not increased was supported by population-based registry studies. Again, the impact of DMTs, particularly high-efficacy therapies was not accounted for in many of these studies.

In contrast, a Norwegian nationwide cohort study found that, although the risk of female genital organ cancer was not different between wwMS and population-based controls between the years 1953–1995, the risk increased significantly from 1996–2017 (4, 21–35). They hypothesized that this may reflect the introduction of highly effective DMTs over this period (34). Again, the study did not directly explore this hypothesis.

Population-wide cohort studies have shown an increased risk of cervical abnormalities in females with autoimmune conditions including inflammatory bowel disease (IBD), systematic lupus erythematosus (SLE), and rheumatoid arthritis (RA), especially if treated with immunomodulatory therapy (4, 38). However, these conditions are not directly comparable with MS, as the diseases have different risk profiles for cervical cancer and most commonly are treated with different DMTs. While most studies in the MS population have found the incidence of cervical cancer to be equal to or less than the general population (4, 21–33, 35), there is growing concern that long-term exposure to DMTs may increase risk.

## 4. Risk of persistent HPV infection and cervical cancer attributable to disease modifying therapies in wwMS

The treatment of multiple sclerosis has been revolutionized over the past two decades with the introduction of highly effective DMTs (2). These therapies have transformed MS disease trajectories by significantly reducing disease activity, relapse rates and disability progression (37). Natalizumab was the first highly effective DMT to receive US Food and Drug Administration (FDA) approval in 2004, and since then there has been successive introductions of new agents, all of which target the immune system in varying ways.

While the benefit of these therapies should be emphasized, they are not without risk. High-efficacy DMTs have been found to increase the risk of opportunistic infections and, theoretically, can reduce immune surveillance and increase cancer risk (2, 39). There is potential for the impact of DMTs to be significant, given that these medications are often commenced at a young age and continued indefinitely (40).

### 4.1. DMTs and the risk of HPV infection

DMTs increase the risk of opportunistic infections within the MS population (39). However, there is currently insufficient data to draw conclusions on the risk of HPV infection (41).

**Figure 2:**
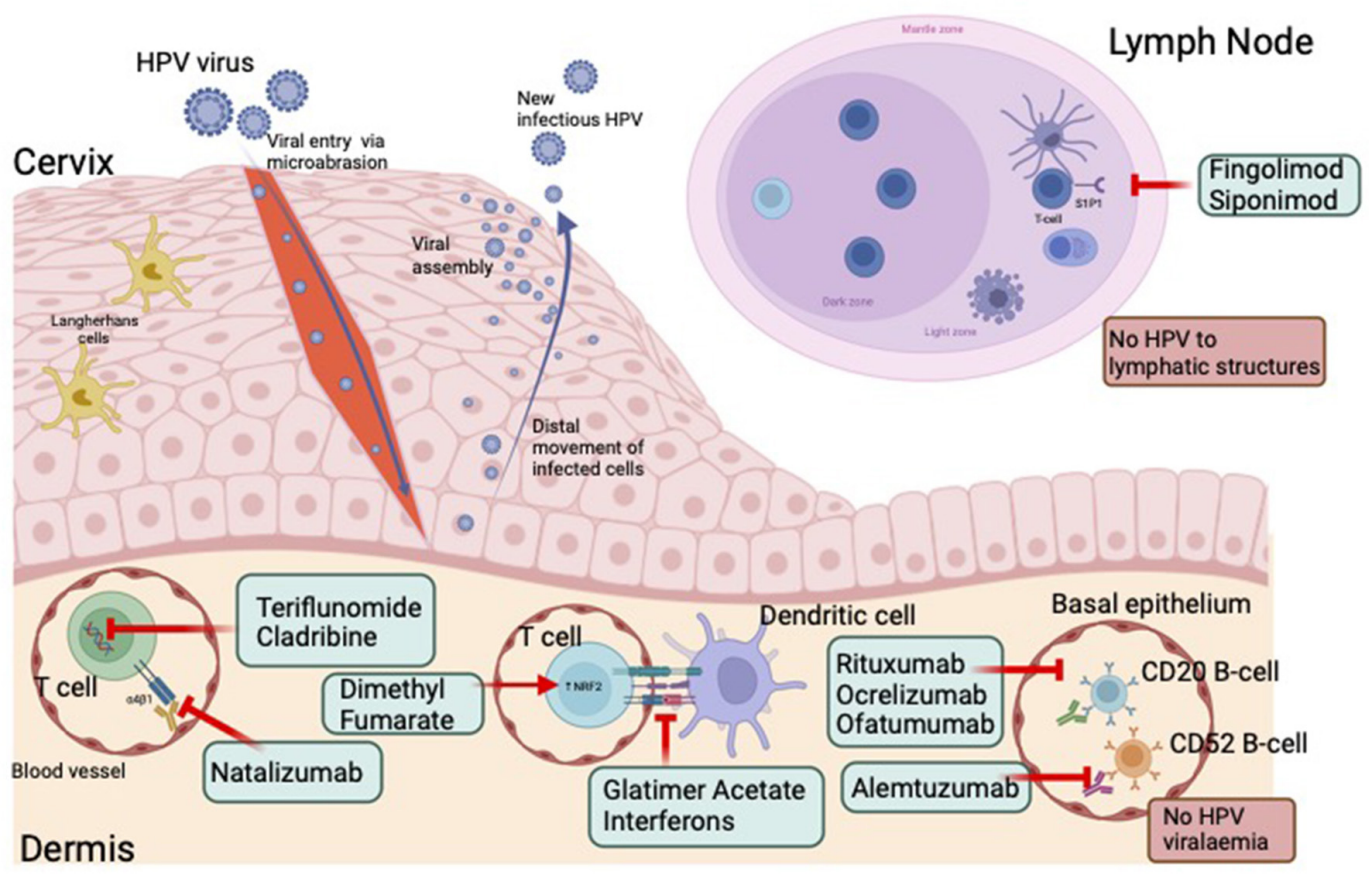
HPV infection, immune response and effect of disease modifying therapies. HPV enters the basal keratinocytes of the cervical epithelium through micro-abrasions. As keratinocytes migrate to the upper epidermis there is increasing viral replication. New infectious HPV is released from the surface of the epithelium. The absence of HPV viraemia or lymphatic infection, results in minimal exposure of HPV to the circulating immune system. DMTs alter the immune response *via* several mechanisms which may further limit immune surveillance and clearance of the virus. HPV, human papillomavirus. Created with BioRender.com.

HPV enters the basal keratinocytes of the cervical epithelium through micro-abrasions (42). As keratinocytes migrate to the upper epidermis there is increasing viral replication (14, 43). New infectious HPV is released from the surface of the epithelium. As HPV is essentially an intracellular pathogen, there is minimal HPV viraemia or lymphatic infection. This results in minimal exposure of HPV to the circulating immune system (14). However, in most cases, activation of the innate and adaptive immune systems does occur, leading to viral clearance in the majority of women (12–14, 44). DMTs alter the immune response *via* several mechanisms which may limit immune surveillance and clearance of the virus (Figure 2). During persistent infections oncogenic types may integrate into the host DNA, disrupting expression of the E6 and E7 viral oncogenes, which may inactivate critical cell cycle checkpoints and increase genetic instability in the host, which over time may lead to cervical cancer (45).

Few studies have investigated the risk of DMTs on HPV infection. Fingolimod has been identified in a small number of case report series as being associated with a risk of HPV infection (39, 46–48). However, this finding has not been validated by higher quality evidence.

### 4.2. Disease modifying therapies and risk of cervical cancer

There is limited data on the effect of DMTs on cervical cancer risk in the MS population (see Supplementary Table 1). At present, studies are limited by sample size, duration of follow-up, difficulty capturing representative population-based samples and complete and accurate data. Additionally, there is likely underreporting of this outcome in part due to under-participation in screening programs in the MS population (6–8, 49). Importantly cervical cancer outcomes may not be captured in the pharmaceutical safety trials, as these trials are often conducted over a shorter duration (1–2 years), and oncogenesis secondary to HPV is known to occur over decades.

Studies to date have found conflicting results regarding the risk of overall cancer associated with DMTs. Most studies have reported no increased risk of cancer (22, 50–55), while others have found an increased cancer risk (22, 50–56). Individual classes of DMTs have also been associated with an increased (26, 57), or a reduced risk of cancer (58). The discrepancy is likely in part explained by study design, with many observational studies grouping therapies, for example as immunomodulating vs. immunosuppressing, in order to improve statistical power. However, many of these therapies have different mechanisms of action and thus different risk profiles. Across studies there is also a lack of uniformity as to which therapies are included. Several studies have included therapies such as azathioprine and cyclophosphamide in the analysis for immunosuppressive therapies (22, 26, 50, 51, 53, 55, 56). These therapies have been widely reported to increase the risk of cancer (59, 60), and are seldom used in the modern-day treatment of MS, thereby likely skewing results.

A further challenge is that patients with MS often switch DMTs throughout their life. This makes quantifying the risk attributable to individual DMTs difficult. Additionally, it poses challenges for calculating the risk for an individual who may have been exposed to several different therapies. The cumulative risk is likely the product of the combination of therapies used, along with the duration of exposure to each therapy (26, 50, 51).

Age at exposure to DMT also likely impacts risk. Similar to the general population, cancer risk in patients with MS increases with advancing age, likely in part due to weakening of the immune system (50, 61). Evidence suggests an additive effect from DMT exposure with several DMTs including cladribine, anti-CD20, alemtuzumab and sphingosine-1-phosphate modulators increasing the risk of malignancy with age (61). However, while aging increases cancer risk more broadly, cervical cancer incidence is highest in younger populations (25–50 years) (62). The impact of aging on cervical cancer risk in the MS population remains unclear but may have implications for long-term malignancy surveillance, and DMT counseling.

Despite the lack of definitive evidence to confirm the role of DMTs in the development of cancer, DMTs including cladribine, fingolimod, natalizumab, alemtuzumab, and ocrelizumab all carry a warning for potential cancer risk (34, 63). It is a requirement that all patients treated with these medications undergo cancer surveillance (40).

The cancer incidence among patients treated in the modern-DMT era was examined in a French study that identified 9,269 patients with MS who had been exposed to DMTs from two population-based disease registers and these were linked to patient records in the French National Cancer Register (26). DMTs were categorized into two groups: immunomodulatory drugs and immunosuppressive drugs. Interferons and glatiramer acetate were considered immunomodulatory therapies, whereas azathioprine, mitoxantrone, mycophenolate mofetil, natalizumab, methotrexate, fingolimod, cladribine and teriflunomide were classified as immunosuppressive therapies. For this analysis, all gynecological cancers, including ovarian, cervical, and uterine were grouped together. WwMS treated with DMTs had a non-significantly increased risk of gynecological cancers (SIR 1.2; CI 0.8–1.9). The risk of “all cancer” was increased if the patient had been exposed to more than three types of immunosuppressive drugs, or more than two types of immunomodulatory drugs. The risk of cancer was increased with increased duration of exposure to DMTs (*P* < 0.001). The mean duration of treatment with immunosuppressive drugs was 4.9 +/- 4.5 years for patients with MS and cancer and 3.6 +/- 4.5 years for patients with MS and no cancer. This observation period is arguably too short to see long-term implications from DMT exposure. Individual DMT analyses, identified only azathioprine and cyclophosphamide as increasing the risk of cancer (RR 1.9, CI 1.7–3.4, *p* = 0.4; RR = 1.9, CI 1.3–2.6, *p* = 0.5, respectively) (26).

## 5. Individual disease modifying therapies and risk of cervical abnormalities

### 5.1. Low efficacy DMTs

#### 5.1.1. Interferons and glatiramer acetate

Glatiramer acetate and the interferon-beta preparations were the first available MS DMTs and were introduced in the 1990's. These therapies are now considered low-efficacy, relative to newer treatments. Interferons exert their effect on the immune system by shifting cytokine profiles toward an anti-inflammatory state and inhibition of leucocyte migration across the blood-brain barrier (64). The mechanism of action of glatiramer acetate is not fully understood, however, it is thought to inhibit T cell responses to several myelin antigens and cause a shift toward Th2 immunity (65, 66).

To date, these therapies have not been associated with an increased cervical cancer incidence in wwMS (67–73).

### 5.2. Moderate to high efficacy DMTs

#### 5.2.1. Dimethyl fumarate

Dimethyl fumarate has been used in the treatment of MS since 2013 due to its proposed anti-inflammatory, immunomodulatory and oxidant properties. While the mechanism by which it exerts its effect is not fully understood, it is thought to suppress the transcription of nuclear factor-kappa B and activate the transcription of the nuclear (erythroid-derived2)-related factor (Nrf2) (74).

DEFINE, a phase three randomized control study found no increased risk of malignancy associated with the use of dimethyl fumarate, one case of cervical cancer was reported in the treatment arm, with no cases reported in the placebo arm (75). This finding was supported by a prospective observational study conducted in Spain over a 5 year period. Again they found no increased risk of malignancy, and only one case of cervical LSIL was reported (76).

#### 5.2.2. Inhibition of lymphocyte migration: Natalizumab and sphingosine-1-phosphate receptor antagonists

##### 5.2.2.1. Natalizumab

Natalizumab was one of the first high-efficacy DMTs and was approved by the FDA in 2004. It is a monoclonal antibody that inhibits alpha4beta1-integrin, thereby preventing T-cells from egressing from the circulation across the blood-brain barrier and into the central nervous system (77). Alpha4beta1 integrins are also located on the surface of the cervix (78) and it is postulated that alpha4beta1 inhibition prevents T-cells from being able to enter the genital mucosa resulting in impaired antimicrobial clearance (79, 80).

A possible association between natalizumab and cervical abnormalities has been described in case reports (79, 81, 82). One case series described four patients treated with natalizumab for 9–45 months who developed high-grade cervical pre-cancer. Three were diagnosed with CIN 2 and one was diagnosed with CIN 3. All four women were positive for HPV (81).

Larger studies have not supported a possible association between natalizumab and cervical cancer (83–86). A Swedish population-based registry study identified no significant risk of pre-cancer (CIN 3+) or cancer in patients treated with natalizumab compared with the general population. However, average duration of follow-up for women treated with natalizumab in this study was 3.94 years. This short duration of surveillance may not have been sufficient to capture cervical cancer outcomes (87).

AFFIRM, a stage 3 randomized, placebo-controlled trial evaluated the safety and efficacy of 627 MS patients treated with natalizumab over a 2-year period. This study identified one case of cervical carcinoma *in situ* in the natalizumab treatment arm (88).

##### 5.2.2.2. Sphingosine 1-P receptor modulators: Fingolimod and siponimod

Fingolimod is a sphingosine-1-phosphate receptor modulator that blocks lymphocyte egress from lymph nodes, limiting the number of circulating T-cells in the peripheral circulation (89). Reductions in the number of circulating T-cells may have implications for immune surveillance, which is a possible mechanism for cancer development (2).

Small case series have found an increase in HPV associated lesions in patients treated with fingolimod (46, 47, 90). A case series of 16 MS patients without a previous history of HPV found that 11 women and 5 men developed HPV lesions following fingolimod initiation (46). The lesions were identified on average 4 years after commencement of fingolimod. Of the nine women who developed cervical abnormalities, five had LSIL and four had HSIL. Oncogenic HPV-16 was identified in three patients. An important limitation of this series was that most patients had been exposed to other immunomodulating therapies prior to the commencement of fingolimod (46).

A Swedish population registry-based cohort study found a borderline significant increased risk of invasive cancer in patients treated with fingolimod compared to the general population (HR 1.53, 95% CI 0.98–2.38) (87). Notably, however, they found no difference in the rates of high-grade cervical pre-cancer (CIN 3) in the fingolimod treated population compared with healthy controls. It is important to note that the average follow-up period was 3.96 years, which may not have been long enough to see an effect on cancers with a long oncogenic lag, such as HPV associated cervical cancer (87).

LONGTERMS, a phase IIIb open-label extension trial, of patients treated with fingolimod for up to 14 years identified seven cases of cervical pre-cancer (0.2%, IR 0.04) (91).

##### 5.2.2.3. Siponimod

Similar to fingolimod, Siponimod is a sphingosine-1-phosphate receptor modulator, which prohibits the egress of immune cells from the lymph nodes into the peripheral circulation (92). It is also thought to exert anti-inflammatory and neuroprotective effects in secondary progressive MS patients (SPMS) (92). It has been FDA approved for both RRMS and SPMS (2).

The stage III randomized, placebo controlled EXPAND trial did not find a difference between the frequency of malignancies in the Siponimod cohort compared to the placebo cohort. There were 11 cases of basal cell carcinoma associated with treatment, however, this did not significantly differ from the placebo arm (93).

#### 5.2.3. Inhibitors of DNA synthesis: Teriflunomide and cladribine

##### 5.2.3.1. Teriflunomide

Teriflunomide inhibits the mitochondrial enzyme dihydroorotate dehydrogenase which is necessary for the synthesis of pyrimidines. This causes inhibition of DNA and RNA synthesis and affects actively dividing cells including immune T and B cells, resulting in less circulating lymphocytes (2). This may reduce immune surveillance, allowing tumorigenesis to go unchecked.

The frequency of malignancy, including cervical cancer, has not been found to be significantly increased in patients treated with teriflunomide (94–96). 9-year follow-up from the TEMSO trial, which evaluated safety and efficacy of teriflunomide, identified one case of cervical carcinoma *in situ* in the teriflunomide treatment arm (97).

##### 5.2.3.2. Cladribine

Cladribine has been newly licensed for the treatment of RRMS and SPMS. It was approved by the FDA in 2019. Cladribine is a nucleoside analog that inhibits DNA synthesis and repair. This results in sustained reduction of circulating B and T lymphocytes (98).

The pharmaceutical safety trials for Cladribine identified one case of cervical carcinoma *in situ* (99, 100). Of note, HPV 16 was identified 3 years prior and the patient was prescribed the higher strength dose of 5.25 mg/kg (101). However, the combined safety data from three previously reported Phase III studies (CLARITY, CLARITY extension and ORACLE-MS), as well as the prospective observational PREMIERE registry for patients prescribed 3.5 mg/kg dose of cladribine, did not identify any cases of cervical pre-cancer or cancer in the cladribine cohort. Two cases of cervical carcinoma *in situ* were identified in the placebo group (102).

#### 5.2.4. Monoclonal antibodies: Rituximab, ocrelizumab, ofatumumab, and alemtuzumab

##### 5.2.4.1. Anti-CD20: Rituximab, ocrelizumab, and ofatumumab

Anti-CD20 monoclonal antibody therapies, including Rituximab, Ocrelizumab, and Ofatumumab, target CD 20 on B cells causing B cell depletion (103). B cells have an important role in the regulatory immunological response to cancer. Tumor metabolites can attract B cells to tumor sites, resulting in detection and lysis of proliferating tumor cells (104). In the absence of B cells, cancer growth may proceed unchecked (2).

Anti-CD20 therapies have not been associated with an increased incidence of invasive cancer (105–109). Class III evidence to support the safety profile of ocrelizumab was obtained by an analysis of pooled safety data from 11 clinical trials including controlled treatment, open-label extension periods of the phase II and III trials and the phase IIIb trials of ocrelizumab in patients with RRMS and SPMS. This data included 5680 patients with MS who received ocrelizumab in clinical trials. There were 18, 218 patient-years of exposure. The rate of malignancy was calculated to be 0.46 (0.37–0.57) per 100 patient-years, which is consistent with the ranges reported in epidemiologic data (110). One case of stage II cervical carcinoma was identified (110).

##### 5.2.4.2. Alemtuzumab

Alemtuzumab is a humanized monoclonal anti-CD-52 antibody which targets B and T lymphocytes, resulting in peripheral lymphocyte depletion, along with a reduction of natural killer cells, dendritic cells, granulocytes and monocytes (111). Pharmaceutical safety trials have not found a statistically significant increased rate of malignancy compared to controls (108, 112–116). One case of cervical cancer has been reported in association with Alemtuzumab (117).

## 6. Other factors that may increase cervical cancer risk in wwMS

### 6.1. HPV vaccination

The development of HPV vaccinations and the introduction of population-wide vaccination programs have become the fundamental pillar of the WHO Global Strategy to accelerate the elimination of cervical cancer as a public health problem (118). There is emerging evidence for cancer prevention in higher Development Index countries with organized population-based vaccination programs (119–123). However, globally rates have yet to fall as the greatest disease burden remains in low-and middle-income countries with the highest populations, where vaccination programs are yet to be implemented (124–126).

HPV vaccines [including the bivalent (HPV 16, 18), quadrivalent (HPV 6, 11, 16, 18), and nonavalent (HPV 6, 11, 16, 18, 31, 33, 45, 52, 58)] are inactive and therefore safe and immunogenic for the immunocompromised population (43, 127–129). Immunocompromised women will likely benefit from vaccination, even if they have already been exposed to the virus, as vaccine induced antibodies can prevent any new infection, reinfection with previously cleared types or spread of infection throughout the genital tract in cases of reactivation of a previously controlled HPV infection. The vaccines will not, however, clear any existing HPV infection, and do not reduce the need for screening (130, 131). The relatively recent introduction of the HPV vaccination program and age criteria (in Australia, women aged younger than 27 in 2007), has meant that many women have not had access to vaccination and would likely benefit from a “catch-up” vaccine. This would preferably be administered prior to the introduction of immunomodulating therapy (46). However, in Australia this is not routinely funded, and is cost-prohibitive for many.

Current Australian guidelines recommend a two-dose schedule of the nonavalent vaccine for immunocompetent people aged 11–14 years (WHO recommends a global primary target age range for HPV vaccination of 9–14 years) (132). Three doses are advised for those who are severely immunocompromised at the time of vaccination and those aged 15 years or older at the time of the first dose (133). To our knowledge, there is no data specifically examining the MS populations response to HPV vaccination. On the basis of extrapolation from other immunocompromised patient populations, the current recommendation is that wwMS on DMTs should be treated as immunocompromised and offered a three-dose schedule (134).

### 6.2. Cervical screening programs

Cervical screening programs remain fundamental for cervical cancer prevention, regardless of HPV vaccination status (128, 135). Screening programs ensure early detection of pre-cancerous cervical abnormalities so that intervention can occur before progression to cervical cancer (42). The current recommendations for the Australian general population is for 5-yearly cervical screening tests (CST) for women aged 25–74 years. A CST comprises a HPV test followed by a liquid based cytology (LBC) test if oncogenic HPV is detected (133).

Many countries with organized population-based screening programs, including Australia, Canada, Sweden and North America, recommend more frequent screening for immunocompromised women compared to the general population (136–141). However, whether wwMS exposed to immunomodulating therapy are included in this recommendation is often unclear. The Cancer Council Australia's Cervical Cancer Screening guideline lists women treated with immunosuppression for autoimmune diseases as a “group that require special consideration.” They state that this group of women could be considered for screening on a 3-yearly interval, but there is no definitive recommendation for wwMS (142). No studies have specifically addressed this question.

WwMS may have reduced participation in preventative health assessments including cervical cancer screening (5–9). The risk of non-participation increases with increasing physical disability (5, 141, 143). Additionally, MS has the potential to impact cognition and mood which may negatively affect a patient's ability to access preventative health care (7).

Other barriers include physical and environmental barriers such as inaccessible medical offices, lack of transportation and difficulty with patient positioning and discomfort; time limitations as procedures such as CSTs may take longer in patients with physical limitations; and attitudinal barriers for both patients and clinicians. Physicians may hold misconceptions about the value of preventative health care due to an incorrect beliefs that physical disability precludes sexual activity, or that wwMS have a reduced life expectancy and therefore preventative screening is not required. WwMS bring their own attitudes about participation in cervical screening programs. Women may have had previous negative experiences with health care visits leading to reluctance to participate.

It is important that clinicians maintain awareness of current guidelines for immunocompromised patients and cervical screening and of new opportunities. For example, Australia's screening program now offers all people with a cervix the option of self-collection (a low-mid vaginal sample is collected by the patient or their clinician to screen for HPV, replacing the need for cervical specimen collection. This test is as accurate for the detection of CIN2+ as a clinician collected sample) (144).

It is likely that any reduced participation in screening will negatively impact on cervical cancer outcomes. A Canadian retrospective cohort study of 6,820 patients with MS found that, although the incidence of cancer was lower in the MS population, patients had a larger tumor size at diagnosis (27). They argued that increased tumor size may reflect a later cancer stage at diagnosis, possibly as a result of reduced engagement with preventative health care.

## 7. Conclusion

There is insufficient data regarding the risk of HPV infection and progression to cervical pre-cancer and cancer in wwMS treated with DMTs. This is particularly evident for women treated with high-efficacy DMTs. Many studies are underpowered to detect cervical pre-cancer and cancer, particularly due to insufficient follow up time to capture this serious outcome. This represents an important knowledge gap in the MS literature and understanding the risk is imperative for the health and safety of wwMS.

Establishing whether DMTs increase the risk of cervical abnormalities will allow individualized counseling for patients regarding their risk profile. It will also guide international primary and secondary prevention strategies including HPV vaccination and cervical screening programs.

More research is needed to identify and address the barriers to participation in vaccination and screening programs for wwMS. Patients and clinicians need to be aware that wwMS are vulnerable to poor participation in these programs so they can better utilize strategies to optimize engagement.

Addressing these factors will significantly impact the rates of cervical abnormalities in the MS population and will help to advance the target of eliminating cervical cancer as a public health problem globally.

## Author contributions

FB: conceptualization, methodology, writing original draft, reviewing and editing, and visualization. JB and YF: writing—reviewing and editing. HB: writing—reviewing and editing and supervision. VJ and AV: conceptualization, writing—reviewing and editing, and supervision. All authors contributed to the article and approved the submitted version.
