# Supplementary material for: Risk of cervical pre-cancer and cancer in women with multiple sclerosis exposed to high efficacy disease modifying therapies
Source: Front Neurol. 2023 Feb 10;14:1119660. doi: 10.3389/fneur.2023.1119660 (PMC9950275; doi:10.3389/fneur.2023.1119660)
Supplement: Supplementary file 1 [file Table_1.docx]

Supplementary Table 1 Studies evaluating the effect of disease modifying therapies on cancer risk and cervical cancer risk

| **Authors** | **Type of study** | | **Location** | | **Participants** | | **Index Period** | | **DMTs** | | **Results**  **Risk/Incidence/n= of overall cancer** | | **Results**  **Risk/Incidence/n(%) of Cervical abnormalities** | | **Interpretation** | |
| --- | --- | --- | --- | --- | --- | --- | --- | --- | --- | --- | --- | --- | --- | --- | --- | --- |
| **Analysis of grouped immunosuppression** | | | | | | | | | | | | | | | | |
| Lebrun et al., 2008(1) | Descriptive study | | France | | 7418 MS patients  5182 women | | 1995 – 2006 | | IM: interferons beta 1a, 1b and GA  IS: AZA, CTX, mitoxantrone and MTX | | **Risk**  IM only  RR 0.8 (95% CI 0.32 – 2.02, p = 0.6357)  IS only  RR 1.96 (0.84-4.61, 0.121)  IM + IS  RR 0.54 (0.15-1.91, 0.3375)  Duration of exposure to IS increased risk  IS/year RR 1.08 (95% CI 1.01-1.16, p = 0.0035) | | **n (%)**  Gynaecological cancer (ovarian, cervix, uterine)  IM only  5 (0.22%)  IS only  3 (0.41%)  IM + IS  2 (0.44%)  (P = 0.75) | | Treatment with IM, IS only and IM + IS exposure did not increase overall cancer risk.  Risk of cancer increased with increasing duration of IS exposure.  Risk of gynaecological cancer not increased in patients treated with IM or IS. | |
| Dugué et al., 2015(2) | Cohort study | | Denmark | | 14403 MS patients | | 1977-2010 | | Antimetabolites (MTX, AZA)  Systemic corticosteroids  Other immunosuppressants | | NR | | **Risk**  Treatment vs non-treatment  HR = 1.0 (95% CI 0.8–1.2)  Antimetabolites  HR 1.1 (95% CI 0.9-1.5)  Corticosteroids HR 0.9 (95% CI 0.7-1.2)  Other IS  HR 0.5 (95% CI 0.2-1.7) | | Treatment did not increase cervical cancer risk. | |
| Ragonese et al.,2017(3) | Cohort study | | Italy | | 531 MS patients | | 1994 - 2011 | | IS: MTX, AZA, CTX | | **Risk**  Cancer  IS treatment  Adjusted HR: 11.05; CI 1.67–73.3; p =0.013 | | No cervical abnormality reported | | Increased risk of cancer in patients exposed to IS therapy. | |
| Moisset et al., 2017(4) | Case-control study | | France | | 1107 MS patients  1568 controls | | 2014-2015 | | IM: Interferonβ, GA, DMF  IS: FTY, NTZ, Mitoxantrone, MTX, AZA  CTX, MMF, Teriflunomide, RIX | | **Risk**  Multivariate analysis  Risk of cancer with IM DMT use  OR 1.22 (95% CI 0.632.35; p=0.55)  Risk of cancer with IS DMT use  OR 0.82 (95% CI 0.33–2.06; p=0.67)  Risk of cancer with IS & IM DMT use  OR 0.78 95% CI 0.331.80; p=0.55) | | **n (%)**  Gynaecological cancers  MS  13 (1.17%)  Controls  28 (2.93%) | | DMT use (immunomodulators immunosuppressants) did not increase the risk of cancer. | |
| D’Amico et al., 2019(5) | Observational study | | Spain | | 1180 MS patients | | 2003-2013 | | IM: Interferonβ, GA  IS: MTZ, Mitoxantrone, FTY, AZA, NTZ | | **Risk**  Cancer  No DMT  RR = 1.03 (CI 95% 0.56–1.41)  No DMT-switch  RR = 1.17 CI 95% (1.02–2.34)  One DMT switch  RR = 1.99 (CI 95% 1.14–3.45)    >/=2 DMT switch  3.38 (CI95% 1.83–6.22) in. | | No cervical abnormality reported | | Higher cancer risk in MS patients switching more than two DMTs. | |
| Gil‑Bernal et al., 2021(6) | Retrospective observational study | | Spain | | 250 MS patients  232 on DMT | | 1981-2019 | | Interferonβ  GA  Teriflunomide  DMF  NTZ  FTY  Alemtuzumab | | **Risk**  Time of Interferonβ use  HR= 0.923 (95% CI 0.873–0.977, p = 0.006)  Time of DMF use  HR = 0.725 (95% CI 0.507-1.036, p = 0.077)  Time of FTY use  HR = 1.219 (95% CI 0.979 – 1.517, p = 0.133) | | No cervical abnormality reported | | Interferonβ and DMF use were protective against neoplasm development. | |
| Mariottini et al., 2022(7) | Case-control study | | Italy | | 661 pwMS (68% exposed to DMTs) | | 2002 - 2018 | | GA  Interferonβ-1A  Interferonβ--1B  DMF  Teriflunomide  FTY  NTZ  Alemtuzumab  Cladribine  Ocrelizumab  Rituximab  Mitoxantrone  AZA  CYP | | **Incidence**  incidence of malignancy  MS cohort: 3.9/ 1000 py (95% CI 3.75–4.15)  Control cohort: 4.1/ 1000py (95% CI 3.76 –4.42) person-years  **SMR**  MS cohort: 2.0/1000 py (95% CI 1.58–2.37)  Control cohort: 2.0 / 1000 py (95% CI 1.58–2.37) | | **n (%)**  Gynaecological cancer  MS  5 (22%)  **Controls**  12 (5%) | | Incidence of cancer and mortality did not differ between pwMS and the general population.  Higher incidence of gynaecological cancer seen in the MS population compared with controls. | |
| **Low efficacy therapies: Interferons and Glatiramer acetate** | | | | | | | | | | | | | | | | |
| Kappos et al., 2006(8)  BENEFIT  NCT00185211 | Phase 3 Placebo-controlled trial | | International multicentre | | 292 Interferonβ-1b  176 Placebo | | 2002-2008 | | Interferonβ-1b | | No cancer reported | | No cervical abnormality reported | |  | |
| O’Connor et al., 2009(9)  BEYOND  NCT00099502 | Phase 3 randomised control trial | | International multicentre | | 1796 Interferonβ-1b  448 GA | | 2003-2005 | | Interferonβ-1b  GA | | No cancer reported | | No cervical abnormality reported | |  | |
| Reder et al., 2010(10) | Cross-sectional observation study | | North America | | 328 Interferonβ-1b | | 1988-2005 | | Interferonβ-1b | | No cancer reported | | No cervical abnormality reported | |  | |
| Bloomgren et al., 2012(11) | Observational study | | North America | | 402,250 patients | | 1996-2011 | | Interferonβ-1a | | Overall cancer incidence NR. Sub analyses performed for individual cancer types did not show increased cancer risk. | | **Incidence**  Cervical cancer  Cumulative reporting rate 0.83 per 100 000 p/years (95% CI 0.4–1.5)  **Risk**  Cervical cancer  Interferonβ-1a vs general population  OR 0.11 (95% CI 0.01 -0.79, p = 0.002)  Interferonβ-1a vs MS Interferonβ-1a non-users  OR 0.22 (95% CI 0.03–1.74, p= 0.082)  Interferonβ-1a vs untreated MS  OR 0.20 (95% CI 0.02–0.71, p = 0.88) | | No significant difference in malignancy prevalence in intramuscular IFNβ-1a users.  Reduced risk of cervical cancer seen in patients treated with Interferonβ-1a compared with the general population.  No difference in risk of cervical cancer seen in MS patients treated with Interferonβ-1a. | |
| Wolinsky et al., 2015(12)  GLACIER  NCT01874145 | Phase 3 Randomised, Parallel-Arm Study | | International multicentre | | 209 GA | | 2013-2014 | | GA | | No cancer reported | | No cervical abnormality reported | |  | |
| **Moderate- or High-Efficacy Therapies** | | | | | | | | | | | | | | | | |
| **Dimethyl-Fumarate** | | | | | | | | | | | | | | | | |
| Gold et al., 2012(13)  DEFINE  NCT00420212 | | Phase 3 randomised, placebo-controlled trial | | International multicentre | | 556 DMF  408 Placebo | | 2007-2011 | | DMF | | **n (%)**  DMF  2 (<1%)  Placebo  2 (<1%) | | **n (%)**  DMF  1(<1%)  Placebo  0 | | No increased risk of malignancy associated with DMF. |
| Gomez-Moreno et al., 2021(14) | | Non-interventionalist, prospective post-marketing study | | Spain | | 886 DMF | | 2014-2019 | | DMF | | **n=**  DMF  7 | | **n=**  LSIL  1 | | No increased risk of malignancy associated with DMF. |
| **Inhibition of Lymphocyte Migration: Natalizumab and Sphingosine-1-Phosphate Receptor Antagonists** | | | | | | | | | | | | | | | | |
| **Natalizumab** | | | | | | | | | | | | | | | | |
| Polman et al., 2006(15)  AFFIRM  NCT00027300 | Phase 3 randomised, placebo-controlled trial | | International multicentre | | 942 MS patients  627 NTZ  315 Placebo | | 2001-2004 | | NTZ | | **n (%)**  NTZ  5 (<1%)  Placebo  1 (<1%) | | **n (%)**  Cervical abnormality  NTZ  NR (<1%)  Placebo  NR (0%)  (p=0.999)  Cervical carcinoma in situ  NTZ  1  Placebo  0 | | Rates of cervical abnormalities were not increased in patients treated with NTZ. | |
| Rolfes et al., 2013(16) | Case report | | Netherlands | | 4 | | <2013 | | NTZ | | NR | | **n =**  Pre-cancer (CIN 2 & 3)  NTZ  4 | | Four cases of cervical pre-cancer reported in association with NTZ. | |
| Durrieu et al., 2018(17) | Case report | | France | | 1 | | 2016 | | NTZ | | NR | | **n =**  Pre-cancer (CIN 3)  NTZ  1 | | One case of cervical pre-cancer seen in association with NTZ. | |
| Wan et al., 2019(18) | Case Report | | Australia | | 1 | | 2007-2009 | | NTZ | | NR | | **n =**  Cervical cancer  NTZ  1 | | Rapid progression LSIL to squamous cell carcinoma of the cervix within 2 years. | |
| Alping et al., 2020(19) | Cohort study | | Sweden | | 6136 MS, 37,801 non-MS controls  1,670 NTZ | | 2011-2017 | | RIX, FTY, NTZ | | **Incidence**  NTZ  IR(/10 000py) = 26.0 (95% CI 15.1–41.6)  Controls  IR 31.0 (95% CI, 27.8–34.4)  **Risk**  NTZ vs controls  HR = 1.01 (95% CI 0.57–1.77) | | **Incidence**  Pre-cancer (CIN 3)  IR (/10 000py) 31.3 (95% CI 17.5-51.6)  **Risk**  HR 1.29 (95% CI 0.71-2.34) | | No difference in risk of invasive cancer between NTZ, and the general population.  No difference in risk of cervical cancer between NTZ, and the general population. | |
| **Fingolimod** | | | | | | | | | | | | | | | | |
| Kappos et al., 2010(20)  FREEDOMS  NCT00289978 | Phase 3 placebo-controlled trial | | International multicentre | | 1033 MS patients  854 FTY 418 Placebo | | 2006-2007 | | FTY | | **n =**  FTY  8  Placebo  10 | | **n =**  Cervical cancer  FTY  0  Placebo  1 | | Incidence of overall cancer and cervical cancer not increased in patients treated with FTY. | |
| Cohen et al., 2010(21)  TRANSFORMS  NCT00340834 | Phase 3  double-blind, parallel group trial | | International multicentre | | 1153 MS patients  857 FTY  435 Interferon Beta-1a | | 2006-2007 | | FTY | | **n =**  FTY  12  Interferon-beta-1a  1 | | No cervical abnormality reported | |  | |
| Calabresi et al., 2014(22)  FREDOMS II  NCT00355134 | Phase 3 placebo-controlled trial | | International multicentre | | 1083 MS patients  728 FTY 355 Placebo. | | 2006-2009 | | FTY | | **n =**  FTY  27  Placebo  8 | | No cervical abnormality reported | |  | |
| Lublin et al., 2016(23)  INFORMS  NCT00731692 | Phase 3 placebo-controlled trial | | International multicentre | | 823 PPMS patients  336 FTY  487 Placebo | | 2008-2011 | | FTY | | **n =**  FTY  26  Placebo  12 | | No cervical abnormality reported | |  | |
| Cohen et al., 2019(24)  LONGTERMS  NCT01201356 | Phase 3b, extension study. | | International multicentre | | 4086 MS patients FTY | | 2010-2017 | | FTY | | **n (%)**  Benign, malignant, and unspecified neoplasms  NR (2.6) | | **Incidence (n=):**  Cervical abnormality  IR 0.04  (7)  **n =**  Cervical cancer  1 | |  | |
| Mhanna et al., 2020(25) | Case series | | France | | 16 MS patients, 11women | | 2019 | | FTY | | NR | | **n (%)**  Cervical abnormalities  9 (56.2%)  (5 LSIL, 4 HSIL) | | Nine cases of cervical abnormalities seen in association with FTY. | |
| Alping et al., 2020(19) | Cohort study | | Sweden | | 6136 patients with MS, 37,801 non-MS controls  1,620 FTY | | 2011-2017 | | RIX, FTY, NTZ | | **Incidence**  FTY  IR (/10 000py) = 44.0 (95% CI 29.2–63.5)  Controls  IR 31.0 (95% CI 27.8–34.4)  **Risk**  FTY vs controls  HR = 1.53 (95% CI = 0.98–2.38) | | **Incidence**  FTY  Cervical abnormality  CIN 3  IR (/10 000py) 39.1 (95% CI 22.8–62.6)  **Risk**  Cervical abnormality  HR 1.63 (95% CI 0.94-2.82) | | No difference in risk of cervical cancer FTY compared with the general population. | |
| **Siponimod** | | | | | | | | | | | | | | | | |
| Kappos et al., 2018(26)  EXPAND  NCT01665144 | Phase 3 randomised placebo-controlled trial | | International multicentre | | 1651 SPMS, 1099 Siponimod, 546 Placebo | | 2013-2015 | | Siponimod | | **n =**  Siponimod  28  Placebo  17 | | No cervical abnormality reported | |  | |
| **Inhibitors of DNA Synthesis: Teriflunomide and Cladribine** | | | | | | | | | | | | | | | | |
| **Teriflunomide** | | | | | | | | | | | | | | | | |
| O’Connor et al., 2011(27)  TEMSO  NCT00134563 | Phase 3 randomised, placebo-controlled trial | | International multicentre | | 1088 MS patients  725 TER  363 Placebo | | 2004-2008 | | TER | | **n =**  TER  1  Placebo  3 | | **n =**  Cervical cancer  TER  1  Placebo  1 | |  | |
| Miller et al., 2014(28)  TOPIC  NCT00622700 | Phase 3  randomised, placebo-controlled trial | | International multicentre | | 618 MS patients  421 TER  197 Placebo | | 2008-2012 | | TER | | No cancer reported | | No cervical abnormality reported | |  | |
| Confavreux et al., 2014(29)  TOWER  NCT00751881 | Phase 3 randomised, placebo-controlled trial | | International multicentre | | 1169 MS patients    780 TER  385 Placebo | | 2008-2011 | | TER | | **n =**  TER  1 | | No cervical abnormality reported | |  | |
| O’Connor et al, 2016(30)  TEMSO Extension  NCT00803049 | Phase 3 extension study | | International multicentre | | 742 TER | | 2008-2013 | | TER | | **Incidence (n)**  0.01 (10) vs general MS population in Sweden 0.11 | | **n =**  Cervical cancer  TER  1 | | Overall incidence cancer comparable to the general MS population in Sweden | |
| **Cladribine** | | | | | | | | | | | | | | | | |
| Giovannoni et al., 2010(31)  CLARITY  NCT00213135 | Phase 3 ramdomised, placebo-controlled trial | | International multicentre | | 1326 RRMS  884 Cladribine  453 Placebo | | 2005-2007 | | Cladribine | | **n (%)**  Cladribine  10 (1.1%)  Placebo  0 (0%) | | **n =**  Cladribine  1 cervical carcinoma in situ  Placebo  0 | |  | |
| Leist et al., 2014(32)  ORACLE-MS  NCT00725985 | Phase 3, ramdomised, placebo-controlled trial | | International multicentre | | 616 patients  410 Cladribine  206 Placebo | | 2008 – 2010 | | Cladribine | | **n =**  Benign, malignant and unspecified neoplasms  Cladribine  4  Placebo  6 | | No cervical abnormality reported | |  | |
| Giovannoni et al., 2018(33)  CLARITY Extension  NCT00641537 | Phase 3b extension study | | International multicentre | | 867  884 Cladribine 453 Placebo | | 2008-2011 | | Cladribine | | **n (%)**  11 (1.4%) | | No cervical abnormality reported | |  | |
| **Monoclonal antibodies: Rituximab, Ocrelizumab and Alemtuzumab** | | | | | | | | | | | | | | | | |
| **Rituximab** | | | | | | | | | | | | | | | | |
| Alping et al., 2020(19) | Cohort study | | Sweden | | 6136 patients with MS, 37,801 non-MS controls  4187 RIX | | 2011-2017 | | RIX, FTY, NTZ | | **Incidence**  RIX  IR (/10 000py) = 34.4 (95% CI 23.7–48.3)  Controls  IR 31.0 (95% CI 27.8–34.4)  **Risk**  RIX vs controls  HR = 0.85, 95% CI = 0.54–1.32) | | **Incidence**  RIX  Cervical abnormalities  CIN 3  IR (/10 000py) 22.3 (95% CI 12.5–36.8)  **Risk**  Cervical abnormalities  HR 1.15 (95% CI 0.66-2.02) | | No difference in risk of cervical cancer between RIX, and the general population. | |
| **Ocrelizumab** | | | | | | | | | | | | | | | | |
| Montablan et al., 2017(34)  ORTORIO | Phase 3 andomised, placebo-controlled trial | | International multicentre | | 732 PPMS patients  488 OCR  244 Placebo | | 2011-2012 | | OCR | | **n (%)**  OCR  11 (2.3%)  Placebo  2 (0.8%) | | **n (%)**  OCR  0 (0%)  Placebo  1 (0.4%) | |  | |
| Hauser et al., 2017(35)  OPERA I  NCT01247324 | Phase 3 randomised trial | | International multicentre | | 821 MS patients  410 OCR  411 Interferon beta | | 2011 – 2013 | | OCR | | **n (%)**  OCR  1 (0.7%)  Interferon beta  1 (0.2%) | | No cervical abnormality reported | |  | |
| Hauser et al., 2017(35)  OPERA II  NCT014123 | Phase 3 randomised trial | | International multicentre | | 835 patients  417 OCR  418 Interferon beta | | 2011- 2013 | | OCR | | **n (%)**  OCR  1 (0.2%)  Interferon beta  1 (0.2%) | | No cervical abnormality reported | | No difference in risk of cervical cancer between OCR and interferon. | |
| Wolinsky et al., 2020(36)  ORTORIO Extension  NCT01194570 | Open-labelled extension study of ORTORIO | | International multicentre | | 527 PPMS patients  367 OCR  160 Placebo | | 2011 – 2019 | | OCR | | **Incidence**  All exposure population  0·91/100py (95% CI 0·61–1·32)  OCR  0.93/100py (95% CI 0·52–1·54)  Placebo  0.27 /100py (95% CI 0·03–0·99) | | No cervical abnormality reported | |  | |
| Hauser et al., 2021(37) | Pooled safety analysis of 11 phase 2 and 3 trials, phase 3b clinical trials | | International multicentre | | 5,680 OCR | | 2008-2020 | | OCR | | **Incidence**  0.46 / 100py (95% CI 0.37–0.57) | | **n =**  1 (cervical carcinoma stage II) | |  | |
| **Alemtuzumab** | | | | | | | | | | | | | | | | |
| Coles et al., 2008(38)  CAMMS223  NCT00050778 | Phase 2 randomised, blinded trial | | International multicentre | | 334 patients  216 Alemtuzumab  118  Interferon 1beta | | 2002-2004 | | Alemtuzumab | | **Incidence (n=)**  Alemtuzumab  0.0044 per person year  (3)  Interferon 1 beta  0.0036 per person year  (1) | | **n =**  Alemtuzumab  1  Interferon-1beta  No | |  | |
| Cohen et al, 2012(39)  CARE MS/ CAMMS323  NCT00530348 | Phase 3 randomised controlled trial | | International multicentre | | 376 Alemtuzumab  187 Interferon beta 1a | | 2007-2009 | | Alemtuzumab | | **n (%)**  Alemtuzumab  (1%)  Interferon beta1a  0 (0%) | | No cervical abnormality reported | |  | |
| Coles et al., 2012(40)  CARE-MS II  CAMMS324  NCT00548405 | Phase 3 randomised controlled trial | | International multicentre | | 596 Alemtuzumab  202 Interferon beta 1a | | 2007-2009 | | Alemtuzumab | | **n =**  Alemtuzumab  5  Interferon beta 1a  2 | | No cervical abnormality reported | |  | |
| Coles et al., 2017(41)  CARE-MS II Extension  NCT00930553 | Extension study of  CARE-MS II | | International multicentre | | 435 Alemtuzumab | | 2009-2014 | | Alemtuzumab | | **Incidence (n=)**  Post-treatment:  EAIR 0.2/ 100 py  (4) | | No cervical abnormality reported | |  | |
| Steingo et al et al., 2020(42)  CAMMS223 Extension  NCT00930553 | Extension study of CAMMS223 | | International multicentre | | 60 Alemtuzumab | | 2002 – 2014 | | Alemtuzumab | | **Incidence (n=)**  EAIR 0.3 /100 py  (2) | | No cervical abnormality reported | |  | |
| **Ofatumumab** | | | | | | | | | | | | | | | | |
| Hauser et al., 2020(43)  ASCLEPIOS I  NCT02792218 | Phase 3 randomised-controlled trial | | International multicentre | | 465 Ofatumumab  462 TER | | 2016-2018 | | Ofatumumab  TER | | **n (%)**  Ofatumumab  1 (0.6%)  TER  3 (0.6%) | | **n =**  Ofatumumab  0  TER  1 | |  | |
| Hauser et al., 2020(43)  ASCLEPIOS II  NCT02792231 | Phase 3 randomised-controlled trial | | International multicentre | | 5481 Ofatumumab  474 TER | | 2016-2018 | | Ofatumumab  TER | | **n (%)**  Ofatumumab  1 (0.4%)  TER  1 (0.2%) | | No cervical abnormality reported | |  | |

Abbreviations: AHR: adjusted hazard ratio, AZA: azathioprine, CI: confidence interval, CIN: cervical intraepithelial neoplasia, CTX: cyclophosphamide, DMF: dimethyl fumerate, DMT: disease modifying therapy, EAIR: exposure-adjusted incidence rate, FTY: fingolimod, GA: glatiramer acetate, HSIL: high grade squamous intraepithelial lesion, HR: hazard ratio, IM: immunomodulatory, IR: incidence rate, IRR: incidence rate ratio, LSIL: low grade squamous intraepithelial lesion, IS: immunosuppressive, IVIG: intravenous immunoglobulin, MMF: mycophenolate mofetil, MS: multiple sclerosis, MTX: methotrexate, NR: not reported, OCR: ocrelizumab, OR: odds ratio, py: person years, RIX: rituximab, RR: relative risk, SIR: Standardised incidence ratio, SMR: Standardised mortality ratio, TER: teriflunomide.

References

1. Lebrun C, Debouverie M, Vermersch P, Clavelou P, Rumbach L, de Seze J, et al. Cancer risk and impact of disease-modifying treatments in patients with multiple sclerosis. Multiple Sclerosis: Clinical and Laboratory Research. 2008 Apr;14(3):399–405.

2. Dugué PA, Rebolj M, Hallas J, Garred P, Lynge E. Risk of cervical cancer in women with autoimmune diseases, in relation with their use of immunosuppressants and screening: population-based cohort study. Int J Cancer. 2015 Mar 15;136(6):E711-719.

3. Ragonese P, Aridon P, Vazzoler G, Mazzola MA, Lo Re V, Lo Re M, et al. Association between multiple sclerosis, cancer risk, and immunosuppressant treatment: a cohort study. BMC Neurol. 2017 Aug 8;17:155.

4. Moisset X, Perié M, Pereira B, Dumont E, Lebrun-Frenay C, Lesage FX, et al. Decreased prevalence of cancer in patients with multiple sclerosis: A case-control study. PLoS One. 2017 Nov 27;12(11):e0188120.

5. D’Amico E, Chisari CG, Arena S, Zanghì A, Toscano S, Lo Fermo S, et al. Cancer Risk and Multiple Sclerosis: Evidence From a Large Italian Cohort. Front Neurol. 2019 Apr 10;10:337.

6. Gil-Bernal R, González-Caballero JL, Espinosa-Rosso R, Gómez-Gómez C. Potential risk of disease modifying therapies on neoplasm development and coadjutant factors in multiple sclerosis outpatients. Sci Rep. 2021 Jun 15;11:12533.

7. Mariottini A, Forci B, Gualdani E, Romoli M, Repice AM, Barilaro A, et al. Incidence of malignant neoplasms and mortality in people affected by multiple sclerosis in the epoch of disease-modifying treatments: A population-based study on Tuscan residents. Mult Scler Relat Disord. 2022 Apr;60:103679.

8. Kappos L, Polman CH, Freedman MS, Edan G, Hartung HP, Miller DH, et al. Treatment with interferon beta-1b delays conversion to clinically definite and McDonald MS in patients with clinically isolated syndromes. Neurology. 2006 Oct 10;67(7):1242–9.

9. O’Connor P, Filippi M, Arnason B, Comi G, Cook S, Goodin D, et al. 250 μg or 500 μg interferon beta-1b versus 20 mg glatiramer acetate in relapsing-remitting multiple sclerosis: a prospective, randomised, multicentre study. The Lancet Neurology. 2009 Oct;8(10):889–97.

10. Reder AT, Ebers GC, Traboulsee A, Li D, Langdon D, Goodin DS, et al. Cross-sectional study assessing long-term safety of interferon- -1b for relapsing-remitting MS. Neurology. 2010 Jun 8;74(23):1877–85.

11. Bloomgren G, Sperling B, Cushing K, Wenten M. Assessment of malignancy risk in patients with multiple sclerosis treated with intramuscular interferon beta-1a: retrospective evaluation using a health insurance claims database and postmarketing surveillance data. Ther Clin Risk Manag. 2012;8:313–21.

12. Wolinsky JS, Borresen TE, Dietrich DW, Wynn D, Sidi Y, Steinerman JR, et al. GLACIER: An open-label, randomized, multicenter study to assess the safety and tolerability of glatiramer acetate 40mg three-times weekly versus 20mg daily in patients with relapsing-remitting multiple sclerosis. Multiple Sclerosis and Related Disorders. 2015 Jul;4(4):370–6.

13. Gold R, Kappos L, Arnold DL, Bar-Or A, Giovannoni G, Selmaj K, et al. Placebo-Controlled Phase 3 Study of Oral BG-12 for Relapsing Multiple Sclerosis. New England Journal of Medicine. 2012 Sep 20;367(12):1098–107.

14. Gómez-Moreno M, Sánchez-Seco VG, Moreno-García S, Cámara PS, Sabin-Muñoz J, Ayuso-Peralta L, et al. Cancer diagnosis in a Spanish cohort of multiple sclerosis patients under dimethylfumarate treatment. Multiple Sclerosis and Related Disorders. 2021 Apr 1;49:102747.

15. Polman CH, O’Connor PW, Havrdova E, Hutchinson M, Kappos L, Miller DH, et al. A Randomized, Placebo-Controlled Trial of Natalizumab for Relapsing Multiple Sclerosis. New England Journal of Medicine. 2006 Mar 2;354(9):899–910.

16. Rolfes L, Lokhorst B, Samijn J, van Puijenbroek E. Cervical dysplasia associated with the use of natalizumab. Neth J Med. 2013 Nov;71(9):494–5.

17. Durrieu G, Dardonville Q, Clanet M, Montastruc JL. Cervical dysplasia in a patient with multiple sclerosis treated with natalizumab. Fundamental & Clinical Pharmacology. 2019;33(1):125–6.

18. Wan KM, Oehler MK. Rapid Progression of Low-Grade Cervical Dysplasia into Invasive Cancer during Natalizumab Treatment for Relapsing Remitting Multiple Sclerosis. Case Rep Oncol. 2019 Jan 18;12(1):59–62.

19. Alping P, Askling J, Burman J, Fink K, Fogdell-Hahn A, Gunnarsson M, et al. Cancer Risk for Fingolimod, Natalizumab, and Rituximab in Multiple Sclerosis Patients. Annals of Neurology. 2020;87(5):688–99.

20. Kappos L, Radue EW, O’Connor P, Polman C, Hohlfeld R, Calabresi P, et al. A Placebo-Controlled Trial of Oral Fingolimod in Relapsing Multiple Sclerosis. New England Journal of Medicine. 2010 Feb 4;362(5):387–401.

21. Cohen JA, Barkhof F, Comi G, Hartung HP, Khatri BO, Montalban X, et al. Oral Fingolimod or Intramuscular Interferon for Relapsing Multiple Sclerosis. The New England Journal of Medicine. 2010 Feb 4;362(5):402–15.

22. Calabresi PA, Radue EW, Goodin D, Jeffery D, Rammohan KW, Reder AT, et al. Safety and efficacy of fingolimod in patients with relapsing-remitting multiple sclerosis (FREEDOMS II): a double-blind, randomised, placebo-controlled, phase 3 trial. The Lancet Neurology. 2014 Jun 1;13(6):545–56.

23. Lublin F, Miller DH, Freedman MS, Cree BAC, Wolinsky JS, Weiner H, et al. Oral fingolimod in primary progressive multiple sclerosis (INFORMS): a phase 3, randomised, double-blind, placebo-controlled trial. The Lancet. 2016 Mar 12;387(10023):1075–84.

24. Cohen JA, Tenenbaum N, Bhatt A, Zhang Y, Kappos L. Extended treatment with fingolimod for relapsing multiple sclerosis: the 14-year LONGTERMS study results. Ther Adv Neurol Disord. 2019 Sep 25;12:1756286419878324.

25. Mhanna E, Nouchi A, Louapre C, De Paz R, Heinzlef O, Bodini B, et al. Human papillomavirus lesions in 16 MS patients treated with fingolimod: Outcomes and vaccination. Mult Scler. 2021 Oct 1;27(11):1794–8.

26. Kappos L, Bar-Or A, Cree BAC, Fox RJ, Giovannoni G, Gold R, et al. Siponimod versus placebo in secondary progressive multiple sclerosis (EXPAND): a double-blind, randomised, phase 3 study. The Lancet. 2018 Mar 31;391(10127):1263–73.

27. O’Connor P, Lublin F, Wolinsky J, Comi G, Kappos L, Freedman M, et al. Effect of teriflunomide on relapses leading to healthcare resource use: results from the TEMSO study. In 2011. p. S95-.

28. Miller AE, Wolinsky JS, Kappos L, Comi G, Freedman MS, Olsson TP, et al. Oral teriflunomide for patients with a first clinical episode suggestive of multiple sclerosis (TOPIC): a randomised, double-blind, placebo-controlled, phase 3 trial. The Lancet Neurology. 2014 Oct;13(10):977–86.

29. Confavreux C, O’Connor P, Comi G, Freedman MS, Miller AE, Olsson TP, et al. Oral teriflunomide for patients with relapsing multiple sclerosis (TOWER): a randomised, double-blind, placebo-controlled, phase 3 trial. The Lancet Neurology. 2014 Mar;13(3):247–56.

30. O’Connor P, Comi G, Freedman MS, Miller AE, Kappos L, Bouchard JP, et al. Long-term safety and efficacy of teriflunomide: Nine-year follow-up of the randomized TEMSO study. Neurology. 2016 Mar 8;86(10):920–30.

31. Leist T, Cook S, Comi G, Montalban X, Giovannoni G, Nolting A, et al. Long-term safety data from the cladribine tablets clinical development program in multiple sclerosis. Mult Scler Relat Disord. 2020 Nov;46:102572.

32. Leist TP, Comi G, Cree BAC, Coyle PK, Freedman MS, Hartung HP, et al. Effect of oral cladribine on time to conversion to clinically definite multiple sclerosis in patients with a first demyelinating event (ORACLE MS): a phase 3 randomised trial. The Lancet Neurology. 2014 Mar 1;13(3):257–67.

33. Giovannoni G, Soelberg Sorensen P, Cook S, Rammohan K, Rieckmann P, Comi G, et al. Safety and efficacy of cladribine tablets in patients with relapsing–remitting multiple sclerosis: Results from the randomized extension trial of the CLARITY study. Mult Scler. 2018 Oct 1;24(12):1594–604.

34. Montalban X, Hauser SL, Kappos L, Arnold DL, Bar-Or A, Comi G, et al. Ocrelizumab versus Placebo in Primary Progressive Multiple Sclerosis. New England Journal of Medicine. 2017 Jan 19;376(3):209–20.

35. Hauser SL, Bar-Or A, Comi G, Giovannoni G, Hartung HP, Hemmer B, et al. Ocrelizumab versus Interferon Beta-1a in Relapsing Multiple Sclerosis. N Engl J Med. 2017 Jan 19;376(3):221–34.

36. Wolinsky JS, Arnold DL, Brochet B, Hartung HP, Montalban X, Naismith RT, et al. Long-term follow-up from the ORATORIO trial of ocrelizumab for primary progressive multiple sclerosis: a post-hoc analysis from the ongoing open-label extension of the randomised, placebo-controlled, phase 3 trial. The Lancet Neurology. 2020 Dec 1;19(12):998–1009.

37. Hauser SL, Kappos L, Montalban X, Craveiro L, Chognot C, Hughes R, et al. Safety of Ocrelizumab in Patients With Relapsing and Primary Progressive Multiple Sclerosis. Neurology. 2021 Oct 19;97(16):e1546–59.

38. CAMMS223 Trial Investigators, Coles AJ, Compston DAS, Selmaj KW, Lake SL, Moran S, et al. Alemtuzumab vs. interferon beta-1a in early multiple sclerosis. N Engl J Med. 2008 Oct 23;359(17):1786–801.

39. Cohen JA, Coles AJ, Arnold DL, Confavreux C, Fox EJ, Hartung HP, et al. Alemtuzumab versus interferon beta 1a as first-line treatment for patients with relapsing-remitting multiple sclerosis: a randomised controlled phase 3 trial. The Lancet. 2012 Nov 24;380(9856):1819–28.

40. Coles AJ, Twyman CL, Arnold DL, Cohen JA, Confavreux C, Fox EJ, et al. Alemtuzumab for patients with relapsing multiple sclerosis after disease-modifying therapy: a randomised controlled phase 3 trial. The Lancet. 2012 Nov 24;380(9856):1829–39.

41. Coles AJ, Cohen JA, Fox EJ, Giovannoni G, Hartung HP, Havrdova E, et al. Alemtuzumab CARE-MS II 5-year follow-up: Efficacy and safety findings. Neurology. 2017 Sep 12;89(11):1117–26.

42. Steingo B, Al Malik Y, Bass AD, Berkovich R, Carraro M, Fernández Ó, et al. Long-term efficacy and safety of alemtuzumab in patients with RRMS: 12-year follow-up of CAMMS223. J Neurol. 2020 Nov;267(11):3343–53.

43. Hauser SL, Bar-Or A, Cohen JA, Comi G, Correale J, Coyle PK, et al. Ofatumumab versus Teriflunomide in Multiple Sclerosis. N Engl J Med. 2020 Aug 6;383(6):546–57.
